# Early Pregnancy in Jennies in the Caribbean: Corpus Luteum Development and Progesterone Production, Uterine and Embryo Dynamics, Conceptus Growth and Maturation

**DOI:** 10.3390/ani12020127

**Published:** 2022-01-06

**Authors:** Lorenzo G. T. M. Segabinazzi, Brandy N. Roberts, Erik W. Peterson, Rachael Ambrosia, Don Bergfelt, Juan Samper, Hilari French, Robert O. Gilbert

**Affiliations:** Department of Clinical Sciences, Ross University School of Veterinary Medicine, Basseterre P.O. Box 334, Saint Kitts and Nevis; lgseg@hotmail.com (L.G.T.M.S.); brandi.roberts.dvm@gmail.com (B.N.R.); erpeterson@rossvet.edu.kn (E.W.P.); rachaelambrosia@students.rossu.edu (R.A.); dbergfelt@rossvet.edu.kn (D.B.); jsamper@qf.org.qa (J.S.); hfrench@rossu.edu (H.F.)

**Keywords:** donkey, embryo, diestrus, progesterone, estradiol, prostaglandin, maternal recognition, pregnancy

## Abstract

**Simple Summary:**

An understanding of the basic mechanisms of reproduction in donkeys is essential, for several reasons. Some donkey breeds are threatened or endangered, and efforts to save these species depend on improved knowledge of reproductive processes. In some parts of the world, donkeys continue to be valued for purposes of work, recreation, or even meat or milk production, as well as the breeding of mules, and reproduction is essential to maintain suitable populations. In others, donkey populations have become feral and represent a nuisance or even a danger to human populations, and improved contraceptive methods are required. Whether for enhancing or inhibiting reproduction, species-specific information is valuable. While the mare has been extensively studied, few studies have explored early pregnancy in jennies. Therefore, this study characterized early embryo development and differences in progesterone profile and changes in the corpus luteum between pregnant and non-pregnant jennies.

**Abstract:**

We aimed to characterize early embryo development and changes in corpus luteum (CL) development and progesterone profile in pregnant vs. non-pregnant jennies. Eight jennies were enrolled in the study. In the first two cycles, the jennies were monitored by transrectal ultrasonography and had blood harvested for hormone profile assay. In the third cycle, jennies were bred by a jack of proven fertility. Jennies were then monitored and sampled for up to 30 days of pregnancy. Data were evaluated by random-effects multiple linear regression, and correlations were expressed as Pearson’s correlation coefficient. Progesterone concentration rose rapidly from ovulation (D0) until D7, plateaued until D12–14, then precipitously declined between D14 and 15, remaining low until the next ovulation in non-pregnant cycles. In the pregnant jennies, the progesterone concentration rose to maximal concentrations on D7–11, being higher at this stage than in non-pregnant cycles, then declined gradually up to D30. In all cycles, the volume of the CL increased steadily until D6, when it plateaued in pregnant jennies. For non-pregnant jennies, CL volume decreased slowly from D6 to D11 and then had a faster drop. Uterine tone increased following ovulation, becoming turgid around the day of embryo fixation (D15.0 ± 0.9). An embryonic vesicle (EV) was first detected on D9.3 ± 0.5 (2.4 ± 0.5 mm). The EV remained spherical until D18.6 ± 1.4. The embryo proper was first detected ventrally in the vesicle on D20.8 ± 1.1 and the embryonic heartbeat by D22.0 ± 0.9. The allantoic sac was identified at D24.0 ± 0.9, and at D30, the allantoic sac filled the ventral half of the EV. This study provides evidence that higher cumulative concentrations of progesterone are correlated to size of the EV, and there were changes in the luteal dynamics and progesterone profiles in pregnant vs. non-pregnant jennies.

## 1. Introduction

During particular life phases, such as pregnancy and lactation, female animals have to face physiological demands and adaptation. Despite the action of homeostatic mechanisms to maintain blood parameters within physiologic levels, changes in metabolites and hormones occur as a result of increased metabolic demands in pregnant animals. These changes are not necessarily indicative of disease, but make animals physiologically unstable and more susceptible to a number of metabolic diseases at this stage than during other life periods, compromising health status, welfare, and productivity. Many papers are currently available on this topic, and, while the mare has been extensively studied [1,2,3,4,5], few studies have explored early pregnancy in jennies [6,7,8,9]. The published literature focuses on the significant differences between jennies, mares, and ponies, but although one study has described some characteristics of early pregnancy in jennies [7]; none fully define the daily progression of jennies’ early pregnancy. In horses, the use of ultrasonography to evaluate pregnancy development and fetal wellbeing has been well described, since 1980 [10,11], and endocrinology during gestation has been extensively studied in mares [12,13]. However, there is a lack of information regarding jennies’ pregnancy, and horse guidelines are often applied for this species [14].

The diagnosis of early pregnancy and evaluation of the conceptus development by ultrasound is valuable in equine breeding programs. The knowledge of endocrinology is an essential step in understanding reproductive processes in farm animals. Few studies have highlighted the characteristics of jennies’ early pregnancy [6,7,8,9]. Progesterone secreted by the primary corpus luteum (CL) is essential for supporting early pregnancy in mammals [15,16,17]. Although progesterone is the hormone most frequently studied and evaluated during pregnancy in mares, relatively little is known about the progesterone profiles in early pregnant jennies [6,8].

An understanding of the basic mechanisms of reproduction in donkeys is important for several reasons. Some donkey breeds are threatened or endangered [18], and efforts to save these species depend on improved knowledge of reproductive processes. In addition, in some parts of the world, donkeys continue to be valued for purposes of work, recreation, to produce mules, or even to produce meat and milk [18,19,20]. In others, donkey populations have become feral and represent a nuisance, or even a danger, to human populations [19], and improved contraceptive methods are required. Whether for enhancing or inhibiting reproduction, species-specific information is a valuable method to maintain suitable populations of these animals [21].

Therefore, the goal of this study was to provide a comprehensive report of the early conceptus in jennies, focusing on the embryonic vesicle (EV), from the first detection to Day 30 of pregnancy (ovulation = Day 0), using transrectal ultrasonography. In addition, this study aimed to explore the differences in circulating progesterone profiles in non-pregnant vs. pregnant jennies and correlate them to the CL volume and size of the EV. More specifically, this study aimed to define the following characteristics: progression of uterine and cervical tone; uterine diameter accompanying pregnancy; ability to detect EV, mobility patterns of the EV; day of fixation; morphological changes; daily growth rate, and identification of embryo proper, embryonic heartbeat, and allantoic sac; as well as knowledge of CL dynamics and progesterone profile associated with the first 30 days of pregnancy in donkey species.

## 2. Materials and Methods

This study was reviewed and approved by the Institutional Animal Care and Use Committee of the Ross University School of Veterinary Medicine under protocol #17.04.16. The study was conducted from January to August 2017 at Ross University School of Veterinary Medicine (RUSVM), Basseterre, St. Kitts, West Indies (17°18′ N 62°44′ W). Eight healthy Caribbean jennies, aged 3 to 12 (±5)-years-old and weighing 100–140 kg, were enrolled in this study. Jennies were housed in outdoor grass paddocks, under natural light, fed with freshly-cut New Guinea grass (*Megathyrsus maximus*) and senior pelleted grain daily, and allowed constant access to fresh water and a trace-mineral salt block. All jennies had previously carried pregnancies to term and were normally cycling before the start of the study, as evidenced follicular growth, ovulation, and CL development, as assessed by ultrasonography.

### 2.1. Study Design

Initially, two estrous cycles of each jenny were monitored for assessment of progesterone profile and CL dynamics during non-pregnant cycles. Thereafter, jennies were bred by a jack of proven fertility, to determine the same parameters, uterine dynamics, and development of the conceptus during the first 30 days of pregnancy.

During the non-pregnant cycles, jennies were monitored by transrectal palpation and ultrasonography (Exago, IMV Imaging, Rochester, MN, USA; 5 MHz linear transducer) daily, and blood samples were obtained every other day from alternating jugular veins via 20 G vacutainer needles. After observation of two consecutive cycles, a jack of proven fertility was used to tease jennies daily, and an estrus score of 0–3 was assigned: 0, aggressively not interested; 1, indifferent; 2, mouth clapping and ear pinning; and 3, mouth clapping, ear pinning, urinating, kicking [20]. Jennies assigned as score 2 or 3 were evaluated by transrectal ultrasound daily. Jennies with signs of estrus and a dominant follicle of >25 mm diameter were placed in a pen and allowed to breed with the jack. Breeding occurred once daily until ovulation was detected by transrectal ultrasonography (D0). Ovulation was defined as the first day there was no longer a dominant follicle and a CL was present. The side of ovulation and the diameter (height × width) of the preovulatory follicle (POF) was recorded one day before the ovulation was diagnosed.

Uterine and cervical tone (0 to 3; 0: flaccid and 3: turgid), endometrial edema (0, no edema; 1, mild edema; 2, moderate edema; 3, evident edema; 4, exacerbated edema) [22], and right and left uterine horn diameters were recorded between the detection of ovulation and 30 days of pregnancy. Cross-sectional measurements of the uterine horns and CL were obtained using internal calipers. The longest dimension and widest perpendicular dimension of the CL and central anechoic cavity were measured. Corpus luteum and central cavity volume were calculated according to the formula for the volume of an ellipsoid, 4/3.π.a.b.c, where a = half of the longest dimension and b = c = radius of the ‘waist’ or widest part of the CL [23]. For CLs with central cavities, volume was calculated after subtraction of the volume of the central cavity.

**Figure 1:**
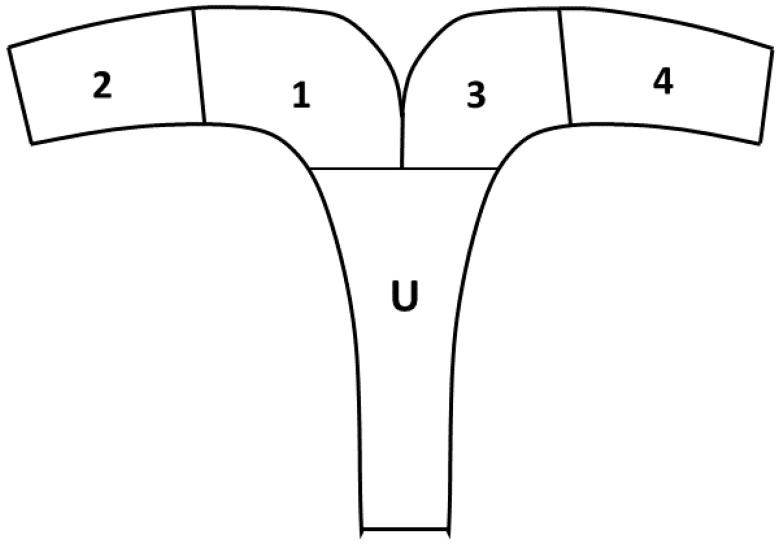
Model for evaluation of embryo mobility in the uterus. U, uterine body; 1, caudal left horn; 2, cranial left horn; 3, caudal right horn; 4, cranial right horn.

Once the embryonic vesicle (EV) was identified via ultrasonography, it was measured (maximum cross-sectional height and width) using internal calipers, and location within the uterus was recorded every ten minutes for two hours daily until fixation. The EV position in the uterus was recorded according to a modified protocol described by Ginther (1984) [24]. The uterus was divided into five sections: U, uterine body; 1, caudal right horn; 2, cranial right horn; 3, caudal left horn; and 4, cranial left horn; and the location of the EV was recorded during each exam (Figure 1). The day of fixation was defined as the day the EV ceased movement during the two-hour assessment and remained in the same location during subsequent exams. Once fixation occurred, daily transrectal ultrasound exams continued to monitor the conceptus. Vesicle size and morphology (spherical, oval, or irregular) were recorded. First detection and location of the embryo proper as an echogenic image within the yolk sac was recorded. Using visual inspection, the presence of a fetal heartbeat and allantoic sac were assessed and recorded when detected. On Day 30 of pregnancy, early termination was induced using prostaglandin F2α (dinoprost 5 mg/animal i.m., Lutalyse^®^, Zoetis, Parsippany-Troy Hills, NJ, USA), and the study was concluded.

Pregnancies were lost spontaneously in two jennies. They were rebred on the subsequent ovulation. Both conceived, and the results of the pregnancies that persisted at least until 30 days are reported here. The EV diameters for the failed pregnancies are reported for comparison, but other data from these pregnancies were not used.

### 2.2. Progesterone Analysis

Blood samples were collected into red top (plain) tubes (BD Vacutainer^®^, BD, USA) every other day in the non-pregnant cycles and every day in the pregnant cycle up to 30 days of pregnancy. After collection, blood was allowed to clot overnight at room temperature (22 °C). Serum was removed the following morning and placed in a −80 °C freezer until samples were analyzed. Progesterone was analyzed in serum samples using a commercial enzyme immunoassay kit (Arbor Assay, K025-H5; Ann Arbor, MI, USA). Validation was done to assess possible matrix effects within the assay when used with donkeys. Serum pools were prepared from samples collected from jennies during estrus, diestrus, and early pregnancy. Each pool was subsequently diluted in a serial manner (1:1, 1:2, 1:4, 1:8, 1:16, 1:32) to determine parallelism with the reference standard curve. Four of the six dilutions fell within the linear range of the standard curve, with a CV of 26%, indicative of parallelism [25]. Thereafter, the assay was conducted according to the manufacturer’s protocol, using 50 uL of serum diluted 1:16 (estrous cycle samples) or 1:32 (pregnancy samples) with assay buffer. Progesterone concentrations that exceeded the high range of the reference standard were further diluted and reanalyzed as necessary. Within-assay CV and sensitivity were 17% and 0.05 ng/mL, respectively.

### 2.3. Statistical Analyses

Data are presented as Mean ± SD or SEM, as stipulated. The Gaussian distribution of the data was evaluated using the Kolmogorov–Smirnov normality test, and all variables were normal (*p* > 0.1). The POF, volume of CL, and circulating concentrations of progesterone were compared between cycling and pregnant jennies by random-effects multiple linear regression, with the day after ovulation and pregnancy status treated as fixed effects and jenny as a random effect to account for repeated measures. The repeatability of measurements within jennies (e.g., progesterone concentration from cycle to cycle) was expressed as an intraclass correlation coefficient. Correlations between normally distributed continuous variables (POF, CL volume, P4 concentrations) were expressed as Pearson’s correlation coefficients. Correlation between the side of ovulation and side of fixation was expressed as Spearman’s correlation coefficient. Strong coefficient of correlation was defined as r > 0.68, and moderate 0.36 ≤ r ≤ 0.68, and weak correlation when r < 0.36. For comparing cumulative progesterone exposure for embryos detected on Day 9 vs. Day 10 post-ovulation, a simple t-test was used. All calculations were performed using Stata/IC 15.1 for Windows (64 bit; StataCorp LLC, College Station TX, USA). Significance was set at *p* < 0.05 for all tests.

## 3. Results

### 3.1. Corpus Luteum and Progesterone Profiles

**Figure 2:**
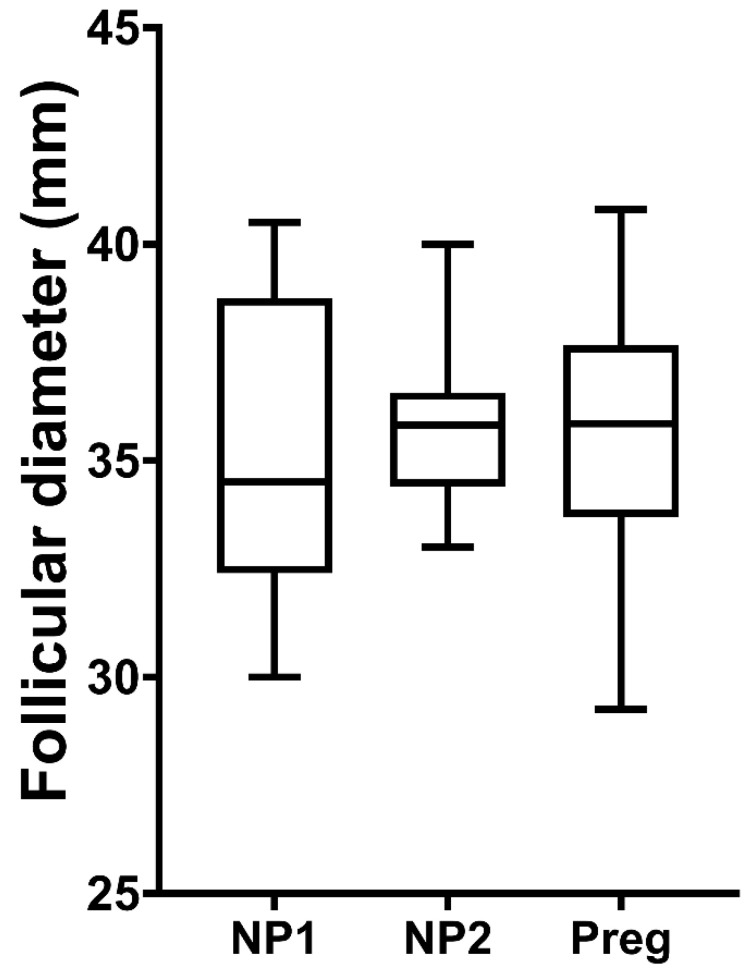
Box plots of the preovulatory follicle diameter in jennies (*n* = 8) in two non-pregnant cycles (NP1 and NP2) and one cycle when the jennies became pregnant after breeding (Preg). The pre-ovulatory diameters were recorded 24 h before the ovulation was diagnosed.

**Figure 3:**
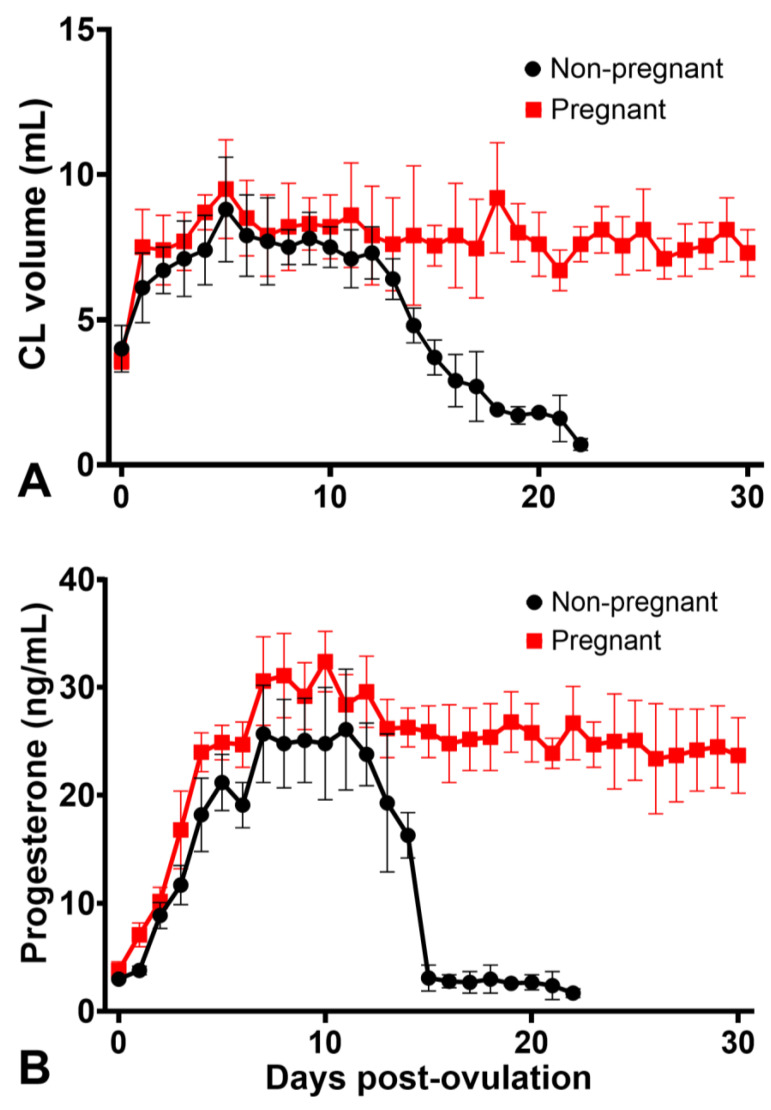
Mean (±SEM) corpus luteum (CL) volume (**A**) and serum progesterone concentrations (**B**) after ovulation (Day 0) for pregnant and non-pregnant jennies.

The preovulatory follicle diameter was similar (*p* > 0.05) in pregnant and non-pregnant cycles (Figure 2). In both groups, the volume of the CL increased steadily until Day 6 (not different between pregnant and non-pregnant jennies; *p* = 0.197), when it plateaued in pregnant jennies (Figure 3A). For non-pregnant jennies, CL volume decreased slowly from Day 6 to Day 11 and, thereafter, declined precipitously. There was a moderate correlation between POF and CL volume up to 10 days post-ovulation in both reproductive states (r = 0.56, *p* < 0.001). The correlation between POF and progesterone concentrations ranged from moderate up to 6 days post-ovulation (r = 0.51; *p* = 0.01), to weak after that period (r = 0.04; *p* = 0.8). Growth in volume of the CL did not differ between pregnant and cycling groups in the first ten days (*p* = 0.123; Table S1).

Mean progesterone concentrations increased rapidly from ovulation until Day 7, plateaued until Day 12, declined gradually until Day 14, and, thereafter, precipitously declined between Days 14 and 15 to baseline until the next ovulation in the non-pregnant cycles (Figure 3B). In the pregnant jennies, progesterone concentration rose to maximal concentrations on Days 7–11, then declined slightly (Day 12), before declining gradually to Day 30, when the study ended (Figure 3B). Overall, mean progesterone concentrations were greater during pregnancy than in non-pregnant cycles. Moreover, when evaluated during the first 5 (*p* < 0.001), 7 (*p* < 0.001) or 10 (*p* < 0.001) days of pregnancy, mean concentrations were higher in pregnant than non-pregnant jennies (Table S2).

Progesterone concentrations were significantly different between jennies and tended to be consistent within jennies; the intraclass correlation coefficient (Day 5–12 after ovulation for non-pregnant cycles) was 0.56 (95% confidence interval: 0.26–0.86), indicating significant repeatability of progesterone concentration within jennies. Progesterone concentrations and CL volume were also moderate related for the first ten days after ovulation (r = 0.51, *p* < 0.001). While progesterone concentrations differed between reproductive status, luteal volume did not differ between non-pregnant and pregnant cycles (*p* = 0.085).

### 3.2. Uterine Dynamics and Embryo Development

All eight jennies conceived on the first breeding. Two jennies exhibited early embryonic loss, one on Day 12 and another on Day 22. They received PGF2α (dinoprost 5 mg/animal i.m.) and were reenrolled into the study after showing signs of estrus. Data from their first pregnancy were omitted from the results comparing various endpoints between pregnant vs. non-pregnant jennies.

**Figure 4:**
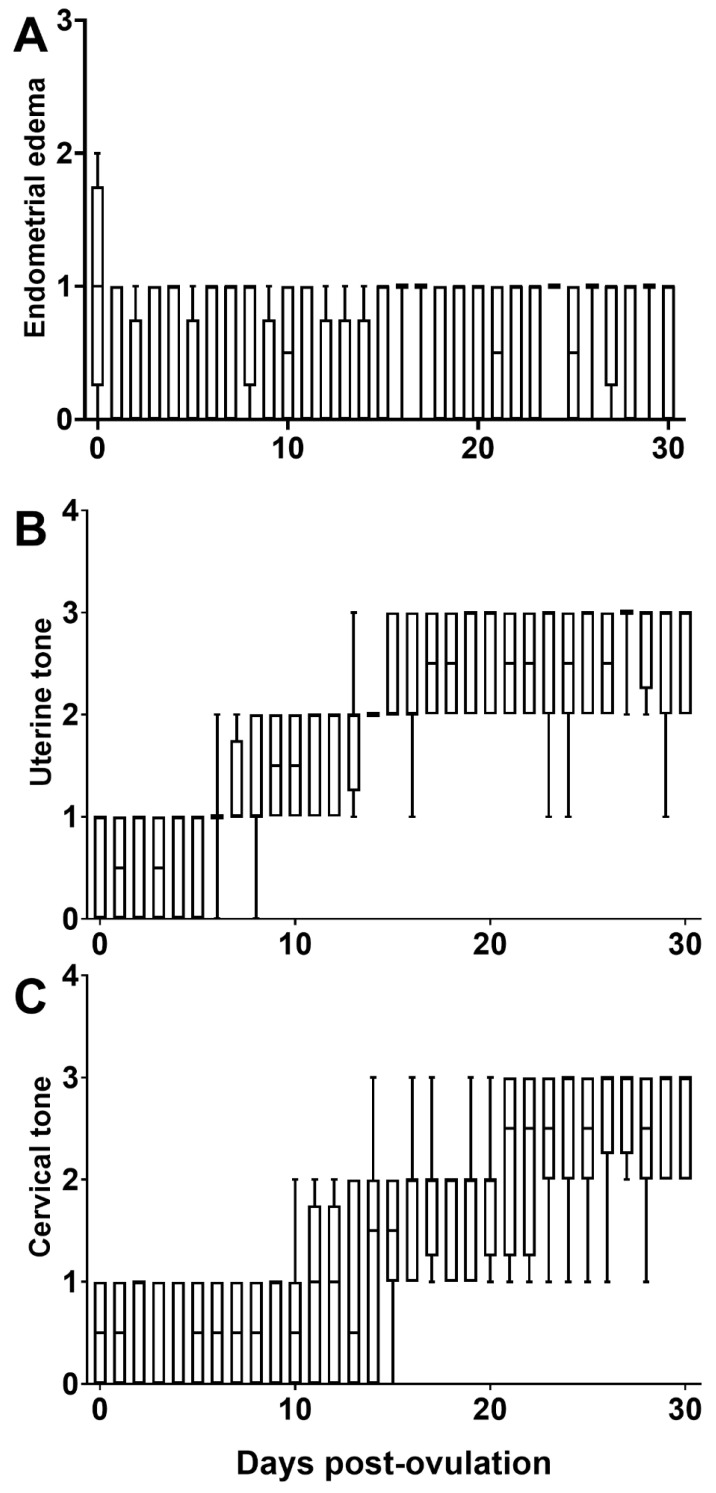
Box plot of the endometrial edema (**A**), uterine (**B**), and cervical tone (**C**) scores in the first 30 days of pregnancy in Caribbean jennies (*n* = 8). Day 0, day of ovulation. Endometrial edema: 0, no edema; 1, mild edema; 2, moderate edema; 3, evident edema; 4, exacerbated edema. Uterine and cervical tone: 0, flaccid and 3, turgid.

Endometrial edema was reduced from three days before ovulation compared to one day after ovulation (Figure S1A). However, the endometrial edema did not show a significant variation between the day when ovulation was detected and 30 days post-ovulation in pregnant jennies (Figure 4A). Uterine tone increased in tonicity following ovulation, becoming turgid one day before fixation or on the day of fixation (15 ± 0.9 days after ovulation; Figure 4B). The cervical tone was noted to increase following ovulation, becoming turgid between Day 12 and 14 (Figure 4C). During estrus up to one day post-ovulation, there were no changes in uterine and cervical tone (Figure S1B,C). The cross-sectional diameters of the uterine horns gradually decreased, from approximately 20 mm post-ovulation to around 15 mm following embryo fixation (Figure S2).

**Figure 5:**
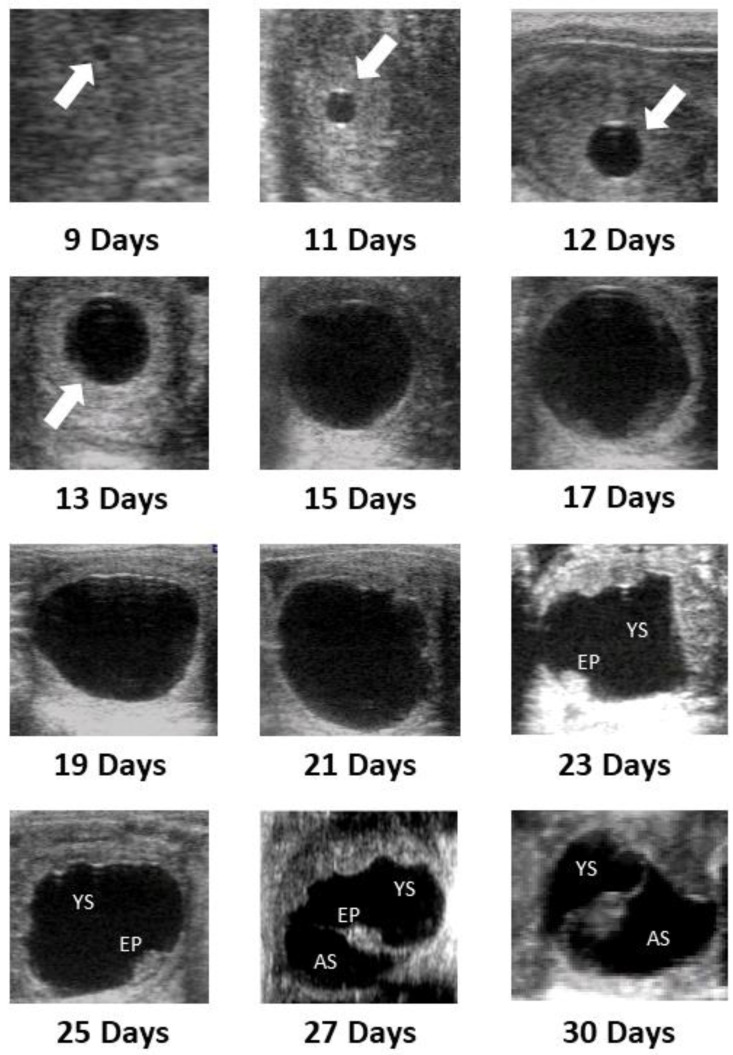
Ultrasound images of the major developmental milestones of the conceptus from Day 9 (day of first detection) to Day 30 of pregnancy in jennies. EV, embryonic vesicle; YS, yolk sac; EP, embryo proper; AS, allantoic sac.

An EV was first detected on Day 9 (6/8) or Day 10 (2/8), with a mean (±SEM) diameter of 2.41 ± 0.45 mm (Table 1). Fixation of the EV occurred on Day 15 ± 0.9 (D13, 1/8; D15, 5/8; and D16, 2/8) with a mean diameter 23.9 ± 1.3 mm one-day preceding fixation. In all jennies, fixation occurred in the caudal segment of the left or right uterine horn. There was no relationship between the side of ovulation and the side of fixation (Spearman’s rho = 0, *p* = 1.0). The EV remained spherical until a mean of 18.6 ± 1.4 days after ovulation. Irregularity in the vesicle shape was first detected on Day 18 and occurred in all vesicles by Day 20 (Table 1). The embryo proper was first detected in the ventral aspect of the vesicle on mean Day 20.8 ± 1.1 (Table 1). Day 21 was the earliest the embryonic heartbeat was identified, with all being detected by Day 23 (mean, 22 ± 0.9). Emergence of the allantoic sac was identified between Days 23 and 26 (mean, 24 ± 0.9), and at Day 30 the allantoic sac replaced the yolk sac by about 50%, with the embryo proper suspended in the center of the vesicle by the apposition of the membranes between the yolk sack and the allantois. Figure 5 illustrates major milestones in the development of the donkey conceptus from Day 9 (day of identification) to Day 30.

Mobility patterns are summarized in Table 2. The EV spent most of the time in the uterine body during Days 9 and 10 of pregnancy. As pregnancy progressed, the EV became more mobile and spent less time in the uterine body. The vesicle’s mobility decreased one day before fixation. During the mobility phase, the mean growth rate of the embryo showed a linear progression, at 3.75 ± 0.58 mm per day. The growth rate plateaued from Days 18 to 28, and the slope was not significantly different from zero (Figure S3). Linear growth resumed on Day 28. In addition, Figure S3 depicts the differences in the EV development between the EV of a jenny that lost pregnancy on Day 22 and jennies that carried a pregnancy up to 30 days.

The increase in mean diameter of the EV from mean day of first detection on Day 9 to Day 18 was not correlated with contemporaneous progesterone (*p* = 0.786). However, EV diameter was correlated (r = 0.64; *p* = 0.000) with cumulative progesterone, calculated as the sum of mean progesterone concentrations from Days 9 to 18, or, roughly, the ‘area under the curve’. Mixed-effects linear regression using the jenny as a random variable and controlling for the day after ovulation showed a significant relationship between EV diameter and cumulative progesterone exposure (Table S3). The cumulative progesterone exposure was 219.5 ± 17.9 ng/mL for those jennies which maintained pregnancy (*n* = 8). Comparatively, for those jennies who spontaneously lost pregnancies (*n* = 2), the cumulative progesterone exposure was 141.7 ± 9.8 ng/mL. The difference between these means approached statistical significance (*p* = 0.056), despite the small numbers.

## 4. Discussion

The POF were similar in the present study between non-pregnant and pregnancy cycles. Similarly, in mares, the POF has been reported as being relatively consistent, which agrees with our results [26,27]. The CL volume was similar up to 10 days post-ovulation in both pregnant and non-pregnant jennies. A moderate correlation between POF and CL volume has also been described in mares [28]. Although a positive correlation between systemic progesterone concentration and POF has been previously reported in mares [29], only moderate to weak correlation was observed in the present study, which is in accordance with another study in mares [28].

The progesterone concentrations in cyclic and pregnant jennies in the present study were similar to previously reported results for jennies [6,30] and those noted for mares [31]. In both cyclic and pregnant jennies, the progesterone concentration peaked between 7 and 10 days after ovulation. After that, the concentration in cyclic jennies declined gradually until Day 15 after ovulation, before falling precipitously between Days 15 and 16. This very abrupt drop in progesterone concentration differs slightly from the observations of Ginther et al. (2006) [32] in mares and Meira et al. [33] in jennies, who reported a steep drop in concentration between Days 14 and 16. It is possible that the tighter degree of synchrony in our observations was coincidental. It is interesting, but consistent with knowledge of luteolysis in mares, that the decline in CL volume was not as dramatic; cessation of progesterone synthesis precedes the physical disappearance of the CL and is mediated first by a reduction in luteal blood flow [34,35,36]. However, luteal blood flow was not assessed in the present study, and the authors cannot comment on this factor in jennies in the Caribbean [37].

The maximum progesterone concentration on Days 7 to 11 observed in pregnant jennies was consistent with previous reports, as was a slight decrease to a plateau maintained until Day 30 after ovulation [6]. However, what is novel is our observation of a higher progesterone concentration in pregnant compared to cycling jennies, through to Day 10 of pregnancy. This difference in progesterone concentration appears not to be mediated by a difference in CL volume, since this did not differ between pregnant and cycling donkeys for the first ten days after ovulation, implying a functional difference mediated by the presence of a conceptus. The current understanding is that there is no difference in circulating progesterone concentration during early pregnancy and in non-pregnant equine cycles. This can be traced back to a 1974 publication examining seven pregnancies and comparing them to seven non-pregnant cycles in different animals [38]. However, when Sevinga et al. examined the characteristics of early pregnancy in Friesian mares [39], they found that pregnant mares had higher progesterone concentration at day 11 after ovulation than non-pregnant mares. They also reported an age difference, with younger mares having higher progesterone concentrations, in both pregnant and non-pregnant states. In a logistic regression analysis, both age and Day 8–9 progesterone concentration were significant predictors of pregnancy. In the study of Sevinga et al. [39], but not in our study, the rate of growth of the CL was also associated with pregnancy. Other authors have previously reported a difference in progesterone concentration on Day 5 after ovulation between pregnant mares and mares that did not become pregnant [26,40]. Given the preponderance of the evidence of a lack of difference in circulating progesterone concentrations in pregnant or cycling mares [41], it is essential to note that the individual variation in progesterone concentrations is considerable. The study by Sevinga et al. [39] used a population drawn from a single breed (Friesian) and controlled for some of the individual variations by including mare age in the analysis. In the study of Canisso et al. [26], the same mares were used in pregnant and non-pregnant cycles. The individual mare was used as a random variable in the multiple linear regression analysis, which is the same approach employed in the present study. We suggest that the effect of pregnancy is obscured by the large variation in progesterone concentration between jennies (or mares) and the relatively repeatable values within jennies if such steps are not taken. Furthermore, although the progesterone concentrations differed between pregnant and cycling jennies, there was no specific cutoff-point or day to distinguish between pregnant or non-pregnant jennies. It is also not clear whether the increased progesterone concentration contributes to pregnancy or is a consequence of it.

Although it is well-established that the primary corpus luteum in horse and pony mares undergoes a functional resurgence later in pregnancy (starting after about 35 days), produces more androgens, estrogens, and progesterone than the cyclic CL of non-pregnancy [41,42], and expresses more significant amounts of steroidogenic acute regulatory protein (StAR) mRNA at this stage [43], comparisons of the primary CL of pregnant and non-pregnant jennies have not been reported. Any evidence that pregnancy or the presence of a conceptus might influence the CL activity is significant, because the mechanism of recognition of pregnancy is not well understood in equids [44], and there has not previously been evidence of such an early effect on luteal steroidogenesis. Apart from the lutealytic mechanism associated with PGF2alpha [45,46], it is evident that some other form of embryo-maternal signaling occurs at an extremely early stage after fertilization. In fact, in equids, uniquely amongst domestic animals, unfertilized oocytes are not transported to the uterus, but remain in the uterine tube indefinitely [47,48], usually to be transported to the uterus passively with the transport of a fertilized zygote at a later cycle. It is now believed that the transport of fertilized oocytes through the uterine tubes to the uterus is mediated, at least in part, by prostaglandins (particularly PGE2) secreted by the zygote itself [49,50]. It is noteworthy that PGE2 is luteotrophic [51,52].

Despite the observation of increased progesterone concentration in the days after ovulation in pregnant mares or jennies, embryo transfer success implies that early embryo presence is not essential to further pregnancy. Indeed, the progesterone concentration in recipient mares before embryo transfer is the same in mares that do or do not retain pregnancy [53].

A further novel finding of the present study is the relationship between embryo diameter and cumulative exposure to progesterone after ovulation. For each day of the study (up to Day 30 after ovulation), the diameter of the EV was positively correlated to the cumulative progesterone exposure. In addition, it is well known that low progesterone levels during early pregnancy can predispose to embryonic losses [54,55]. Ginther [56] reported lower progesterone concentrations at Day 7 after ovulation in mares that suffered pregnancy loss between Days 11 and 15 than in mares remaining pregnant. Moreover, an involvement of progesterone endometrial gene transcription and histotrophic composition at the time of maternal recognition of pregnancy has been reported [57,58]. One manifestation of this variance is the difference in progesterone exposure in those jennies in which the EV was diagnosed at nine days or later. Although this is a novel observation in equids, it is well-established that bovine embryos exposed to higher progesterone concentrations are larger than their contemporaries with less progesterone exposure [59]. This is important because of the transfer of larger equine embryos; controlling for age, embryos with a larger diameter at 12 days post ovulation are more likely to survive to 50 days [60], which, in turn, implies that embryos exposed to higher progesterone concentrations in the early post-ovulation period may have a survival advantage. In the two pregnancy losses observed during the present study, the diameter of the EV dropped below the mean for successful pregnancies before the vesicle disappeared.

Previous studies have demonstrated the jenny’s EV is first detectable on day 10 or 11, once the vesicle has obtained a mean diameter of 4 mm [7,61]. The present study found 75% of vesicles on Day 9 and the remaining on Day 10, with a mean diameter of 2.41 mm at the time of detection. It is suspected that the advancement in ultrasound technology and user skill contributed to identifying EV earlier than previously described. Being able to identify an EV as early as possible is financially beneficial in reproductive operations, as well as helpful in distinguishing between difficulty with conception and early embryonic loss.

The mobility patterns of the EV were similar to the reported data in mares [62,63]. Growth rate and vesicle size were similar to those mentioned in previous studies. Previous studies reported fixation occurring on Day 15 of pregnancy [7,61,64]. Our study demonstrated fixation occurring between Day 13 and Day 16. In 7/8 jennies, the vesicle size one day before fixation was less than 22 mm. Once the EV became greater than 22 mm, fixation occurred. This data suggests that the size of the EV is more critical to fixation than the day of pregnancy. In addition, the uterine tone in 88% of the jennies became turgid one day before fixation, with the remaining 22% becoming turgid on the day of fixation. Cervical tone increased and became turgid during pregnancy; however, there was no consistent pattern in the time of turgidity. In all jennies, there was a notable decrease in uterine horn diameters nearing fixation. The increase in uterine tone and decrease in horn diameter is consistent with the findings of other studies and proved to be beneficial in establishing the appropriate environment for an early pregnancy [64]. Shape of the vesicle was found to be consistent with previous studies in jennies [7,64]. The vesicle retained its spherical shape from the first day of visualization to fixation at around 16 days, but thereafter became irregular. The embryo proper appears to be at the ventral pole around Day 21 after ovulation, transitioning from the yolk sac to the allantoic sac phase. On Day 21–23 of pregnancy, the embryo heartbeat can be detected. The mean day of the first detection of the embryo proper, embryonic heartbeat, and the allantoic sac were consistent with past studies in jennies and mares [7,10,64]. This information helps in evaluating early pregnancies and may guide practitioners in identifying potential fertility issues in jennies that have trouble maintaining early pregnancy.

## 5. Conclusions

This study followed eight pregnancies, from day of identification to Day 30 of pregnancy and provided a comprehensive report of the characteristics of early pregnancy in jennies. This study provides evidence that higher cumulative concentrations of progesterone occurred in the early pregnancy of jennies compared to concentrations in non-pregnant jennies, and this was correlated to size of the EV, which implies earlier communication between the conceptus and luteal steroidogenesis than previously recognized. There were changes in the luteal dynamics and progesterone profiles in pregnancy vs. non-pregnant jennies. The early pregnancy development (up to 30 days) was well described in the present study. This information provides a basis for future studies wanting to increase reproductive efficiency in jennies.

## Figures and Tables

**Table 1:** Days from first ultrasonic detection of the EV developmental characteristics thereafter in eight jennies.

	Day From First Detection	
	Mean ± SEM	Range
Embryonic vesicle	9.25 ± 0.2	9–10
Fixation	15 ± 0.3	13–16
Loss of spherical shape	18.6 ± 0.4	16–20
Embryo proper	20.8 ± 0.4	19–21
Embryonic heartbeat	22 ± 0.3	21–23
Allantoic sac	24.4 ± 0.3	23–26

**Table 2:** Mean (±SD) mobility characteristics of the embryonic vesicle (EV) during a 2-h mobility trial.

Day of Pregnancy	Total Moves	How Many Segments the EV Crossed (Distance Traveled)	Number of Horn Changes	Percentage of Time in the Body
9 (*n* = 5)	3.6 ± 2.0	4.2 ± 2.6	0.8 ± 0.8	58.6%
10 (*n* = 7)	3.6 ± 2.6	3.7 ± 2.9	0.3 ± 0.7	65.6%
11 (*n* = 8)	5.5 ± 2.6	6.5 ± 2.8	1.1 ± 1.1	35.0%
12 (*n* = 8)	5.0 ± 0.9	5.8 ± 1.1	1.1 ± 0.8	41.0%
13 (*n* = 8)	4.8 ± 2.3	5.8 ± 3.0	1.3 ± 1.1	37.0%
14 (*n* = 7)	5.0 ± 1.8	5.6 ± 2.2	0.9 ± 0.8	19.9%
15 (*n* = 7)	0.6 ± 0.9	0.6 ± 0.9	0.1 ± 0.3	2.1%

## Data Availability

The original contributions presented in the study are included in the article/Supplementary Materials, further inquiries can be directed to the corresponding author/s.

## Supplementary Materials

The following are available online at https://www.mdpi.com/article/10.3390/ani12020127/s1, Figure S1. Median and interquartile ranges of endometrial edema (**A**), uterine (**B**), and cervical tone (**C**) of jennies (*n* = 8) during the days from ovulation. Day 0, day of ovulation. Endometrial edema: 0, no edema; 1, mild edema; 2, moderate edema; 3, evident edema; 4, exacerbated edema. Uterine and cervical tone: 0, flaccid and 3, turgid. Different superscripts (a,b) denotes difference between moments (*p* < 0.05). Figure S2. Mean ± SD of the uterine horn (UH) and the embryonic vesicle (EV) diameters during the first 30 days of pregnancy in eight Caribbean jennies. Day of ovulation, day 0. Figure S3. Mean ± SEM diameter of embryonic vesicle from the day of detection (Day 9–10) to Day 30 of pregnancy for embryos surviving at least to 30 days (Normal pregnancy), and for one individual pregnancy that terminated spontaneously 22 days after ovulation (Failed pregnancy). Table S1. Mixed effects linear regression coefficients for CL volume for the first 10 days after ovulation, with the jenny as random variable. Table S2. Mixed effects linear regression for progesterone concentration for first 10 days after ovulation, with the jenny as random variable. Table S3. Mixed effects linear regression coefficients for embryonic vesicle diameter, including the jenny as random variable, up to Day 18 after ovulation.
