# Supplementary material for: Early Pregnancy in Jennies in the Caribbean: Corpus Luteum Development and Progesterone Production, Uterine and Embryo Dynamics, Conceptus Growth and Maturation
Source: Animals (Basel). 2022 Jan 6;12(2):127. doi: 10.3390/ani12020127 (PMC8772573; doi:10.3390/ani12020127)
Supplement: Supplementary file 1 [file animals-12-00127-s001.zip › animals-1508714-supplementary.pdf]

Supplement

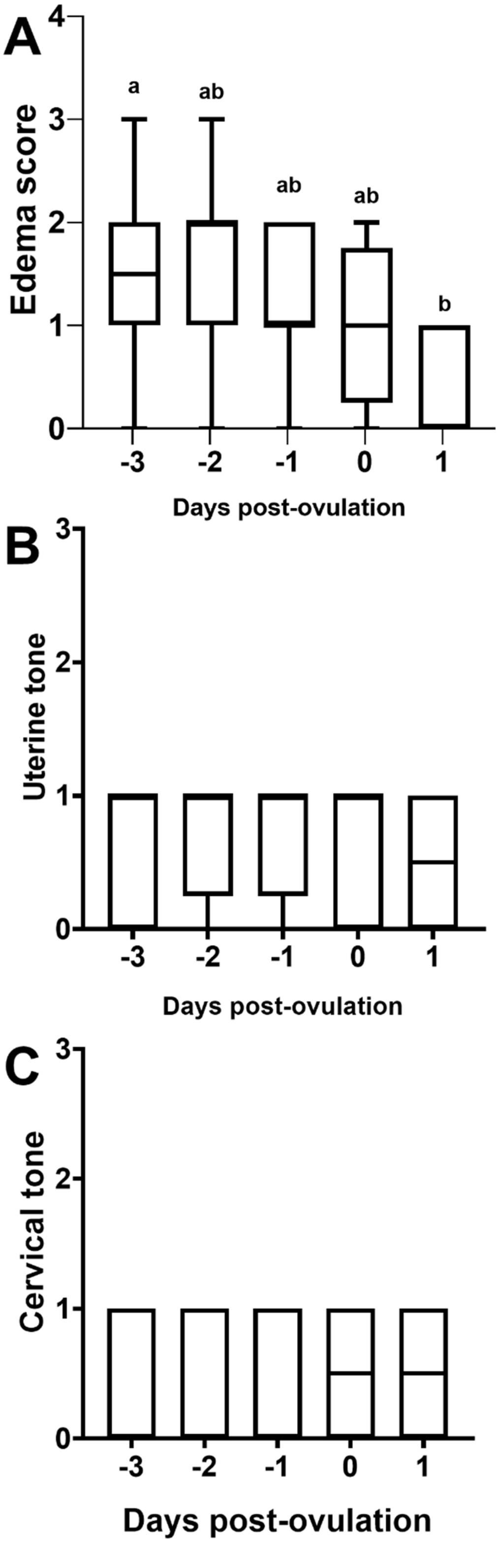

Figure S1. Median and interquartile ranges of endometrial edema (A), uterine (B), and cervical tone (C) of jennies (n = 8) during the days from ovulation. Day 0, day of ovulation. Endometrial edema: 0, no edema; 1, mild edema; 2, moderate edema; 3, evident edema; 4, exacerbated edema. Uterine and cervical tone: 0, flaccid and 3, turgid. Different superscripts ([a,b]) denotes difference between moments (P<0.05).

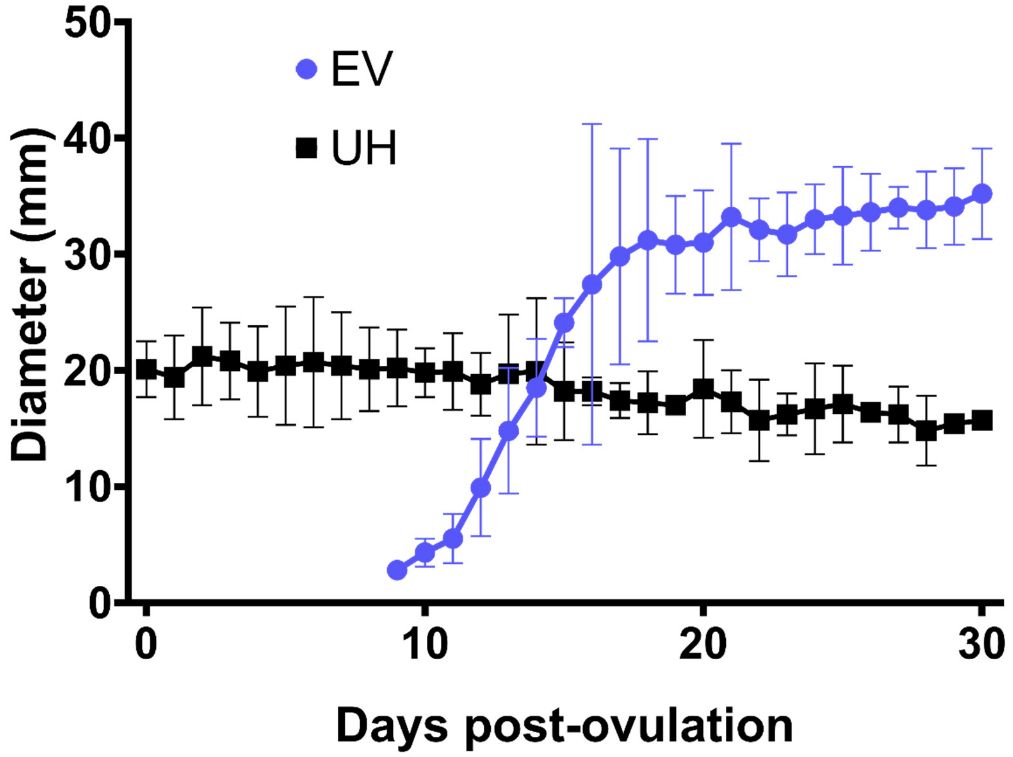

Figure S2. Mean ± SD of the uterine horn (UH) and the embryonic vesicle (EV) diameters during the first 30 days of pregnancy in eight Caribbean jennies. Day of ovulation, day 0.

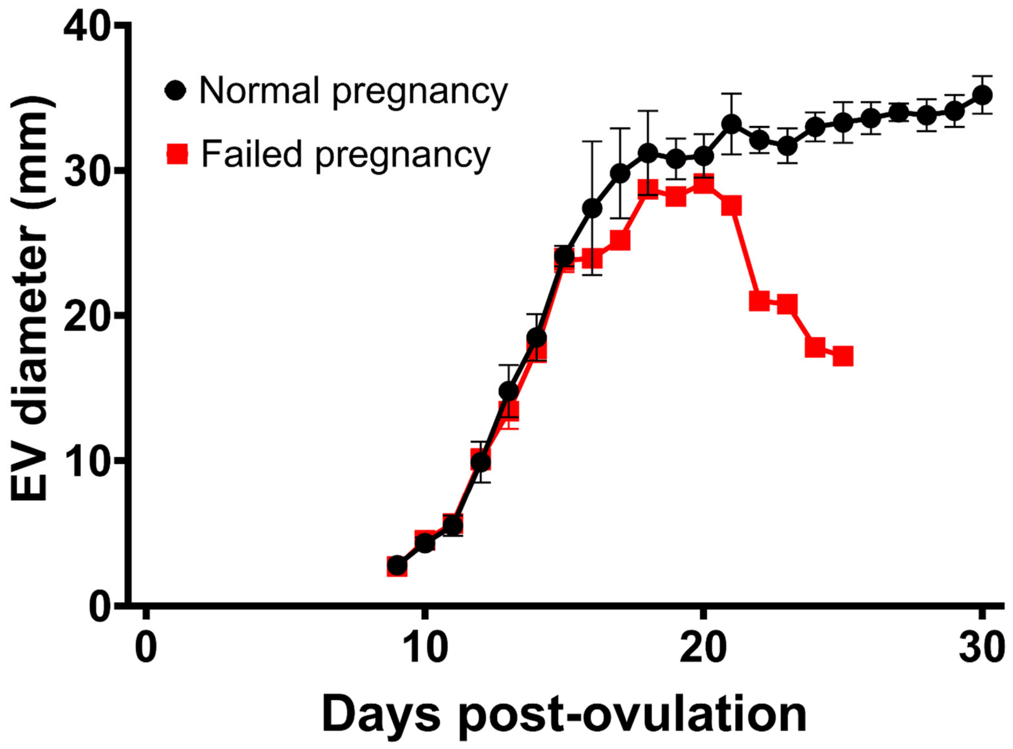

Figure S3. Mean±SEM diameter of embryonic vesicle from the day of detection (Day 9-10) to Day 30 of pregnancy for embryos surviving at least to 30 days (Normal pregnancy), and for one individual pregnancy that terminated spontaneously 22 days after ovulation (Failed pregnancy).

Table S1. Mixed effects linear regression coefficients for CL volume for the first 10 days after ovulation, with jenny as random variable.

Factor	Coefficient	St. Error	z	P>|z|	95% confidence interval	
**Pregnancy**	0.70	0.45	6.2	0.123	-0.19	1.58
**Day**	0.38	0.06	6.2	0.000	0.26	0.50
**Constant**	5.5	0.61	9.01	0.000	4.31	6.70

Table S2. Mixed effects linear regression for progesterone concentration for first 10 days after ovulation, with jenny as random variable.

Factor	Coefficient	St. Error	z	P>|z|	95% confidence interval	
**Pregnancy**	4.25	0.98	4.35	0.000	2.34	6.16
**Day**	2.74	0.14	19.26	0.000	2.46	3.02
**Constant**	3.23	1.91	1.70	0.090	-0.50	6.97

Table S3. Mixed effects linear regression coefficients for embryonic vesicle diameter, including jenny as random variable, up to Day 18 after ovulation.

Factor	Coefficient	St. Error	z	P>|z|	95% confidence interval	
**Day**	2.98	0.22	13.51	0.000	2.55	3.42
**Cum. P4**	0.025	0.007	3.38	0.001	0.011	0.39
**Constant**	-31.99	1.44	-22.27	0.000	-34.82	-29.18
